# Recent Advances in Evolving Computing Paradigms: Cloud, Edge, and Fog Technologies

**DOI:** 10.3390/s22010196

**Published:** 2021-12-28

**Authors:** Nancy A Angel, Dakshanamoorthy Ravindran, P M Durai Raj Vincent, Kathiravan Srinivasan, Yuh-Chung Hu

**Affiliations:** 1 Department of Computer Science, St. Joseph’s College (Autonomous), Bharathidasan University, Tiruchirappalli 620002, India; angelnancy_phdcs@mail.sjctni.edu (N.A.A.); ravindran_cs1@mail.sjctni.edu (D.R.); 2 School of Information Technology and Engineering, Vellore Institute of Technology, Vellore 632014, India; pmvincent@vit.ac.in; 3 School of Computer Science and Engineering, Vellore Institute of Technology, Vellore 632014, India; kathiravan.srinivasan@vit.ac.in; 4 Department of Mechanical and Electromechanical Engineering, National ILan University, Yilan 26047, Taiwan

**Keywords:** cloud computing, edge computing, fog computing, internet-of-things, machine learning

## Abstract

Cloud computing has become integral lately due to the ever-expanding Internet-of-things (IoT) network. It still is and continues to be the best practice for implementing complex computational applications, emphasizing the massive processing of data. However, the cloud falls short due to the critical constraints of novel IoT applications generating vast data, which entails a swift response time with improved privacy. The newest drift is moving computational and storage resources to the edge of the network, involving a decentralized distributed architecture. The data processing and analytics perform at proximity to end-users, and overcome the bottleneck of cloud computing. The trend of deploying machine learning (ML) at the network edge to enhance computing applications and services has gained momentum lately, specifically to reduce latency and energy consumed while optimizing the security and management of resources. There is a need for rigorous research efforts oriented towards developing and implementing machine learning algorithms that deliver the best results in terms of speed, accuracy, storage, and security, with low power consumption. This extensive survey presented on the prominent computing paradigms in practice highlights the latest innovations resulting from the fusion between ML and the evolving computing paradigms and discusses the underlying open research challenges and future prospects.

## 1. Introduction

There has been a significant progression of computing paradigms during recent decades. Cloud computing is perhaps the most well-established, which emerged from the requirement of harnessing “computing as a utility”, enabling the rapid growth of new internet services [1]. The arrival of the Internet of Things (IoT) paved the way for vast data generation, eventually leading to big data [2]. Cloud computing was a hot research area until the widespread use of the Internet of Things disclosed all of the centralized paradigm’s flaws [1]. With cloud-based deployment, cloud data centers manage the analyzing, storing, and decision-making of data. As the data volume along with the velocity surged, transferring the big data brought forth by IoT devices to the cloud became inefficient, owing to bandwidth constraints, and would not meet the time-sensitive and ultra-low latency demands of applications and could raise privacy concerns as well.

The scope of IoT has broadened since its advent and specifies a digital interconnection of devices and objects, capable of procuring and sharing information across platforms for added value [3]. The proliferation of IoT is consorted by an increased capacity, reduced communication cost, and astounding technological development. IoT warrants not just device data management, but also information exchange among multidisciplinary platforms. The huge data procured from numerous smart devices entails sharing to add value and a comprehensive understanding of the concerned domain. With collaborative IoT, heterogeneous domains and settings enable sensors, gateways, and services to collaborate at various levels, enriching the quality of human life while improving business processes.

The IoT ecosystem extends in scale and complexity, encompassing a range of heterogeneous devices that stretch over several layers of IoT architecture. As IoT systems partake in critical infrastructures, they necessitate resilient service operability [4]. IoT applications are disparate, deployed in healthcare, industries, domotics, smart homes, smart cities, smart transportation, etc. The IoT devices are constituted of small, resource-constrained smart objects, ineffective at handling complex tasks, which entails task offloading to distant cloud servers [5]. The limited storage and computing potential forces IoT devices to rely on cloud data centers [6]. This ensues an increased latency, and the intermittent internet connectivity renders IoT devices inept at managing time-critical real-time applications.

Thus, the IoT revolution has steered new research into decentralized models. In this context, edge computing emerged, intending to bring cloud computing capability to the network edge, addressing unfolding issues that cannot be fixed by cloud computing solely, such as latency, bandwidth, and connectivity challenges [7]. Correspondingly, numerous edge computing solutions have been suggested, including Mobile Cloud Computing (MCC) and Mobile Edge Computing (MEC) [8,9]. Fog computing surfaced as one of the highly evolved Edge computing concepts. Fog computing aspires to represent a comprehensive framework, allocating resources in sequence along the cloud to the smart devices [10]. Thus, it is not a mere cloud extension, as it actively engages in synergizing the cloud with IoT. In addition, the requisite for sustainable/green computing that aids in conserving energy is crucial to IoT devices. As IoT devices have energy limitations, it is vital to devise energy-aware solutions into the future [11]. In parallel with technological progress, it is imperative to cut back on the carbon footprint to limit environmental deterioration alongside global warming [12]. The exploration of edge paradigms is at its budding phase, and innovative viewpoints pertaining to these paradigms that arise in literature regularly warrant extensive research [13].

**Figure 1:**
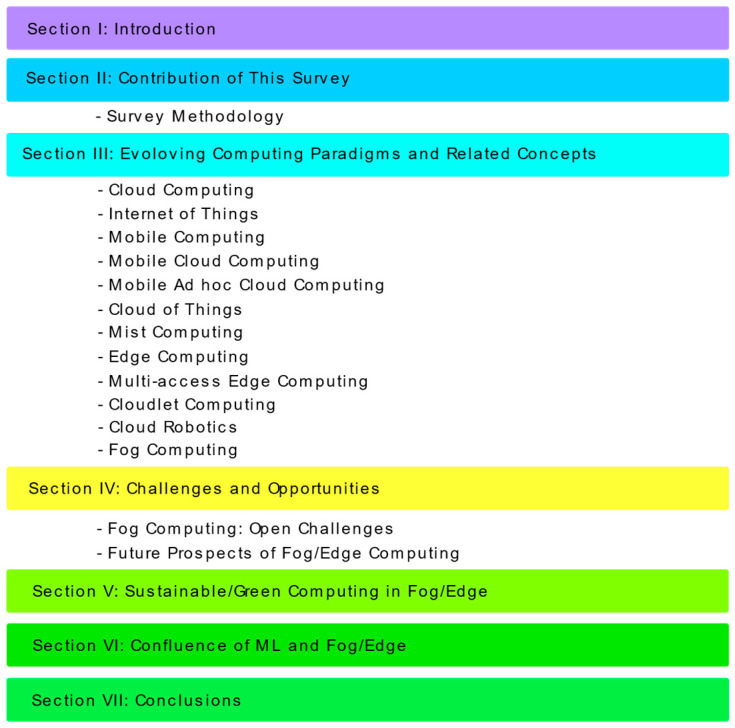
Organization of this survey paper.

Table 1 shows the list of acronyms used in this manuscript. Figure 1 shows the structure of this survey.

## 2. Contribution of This Survey

The contribution of this survey is outlined as follows:

- A comprehensive account of computing paradigms is rendered, especially cloud computing, fog computing, edge computing, and how they are related to other similar paradigms such as mist, cloudlet, MEC, etc.
- A detailed illustration of the motives that instigated the evolution of edge/fog computing and related paradigms is furnished.
- A comparison of cloud, edge, and fog computing paradigms are presented and ML convergence’s significance with fog/edge is discussed.
- A list of challenges and future research directions concerning computing paradigms is devised.

### 2.1. Survey Methodology

We harnessed the Preferred Reporting Items for Systematic Reviews and Meta-Analyses (PRISMA) procedure to systematically choose the articles used in this survey.

#### 2.1.1. Search Strategy and Literature Sources

For this review, articles pertaining to Evolving Computing Paradigms were searched in Google Scholar, ScienceDirect, IEEE Xplore, ACM Digital Library, Wiley Online Library, and Springer databases from January 2009 to January 2022.

The search string used in this study was (“Cloud computing” or “Edge computing”, or “Fog computing” or “Internet-of-Things” or “Machine learning”) and collected 2360 articles.

#### 2.1.2. Inclusion Criteria

The articles written and published in English between January 2009 and January 2022 on Evolving Computing Paradigms were included. This review includes relatively new research.

#### 2.1.3. Elimination Criteria

The articles published in languages other than English, from January 2009, including case reports/case series, opinions, letters to the editor, commentaries, conference abstracts, theses, and dissertations, were excluded from this review.

#### 2.1.4. Results

**Figure 2:**
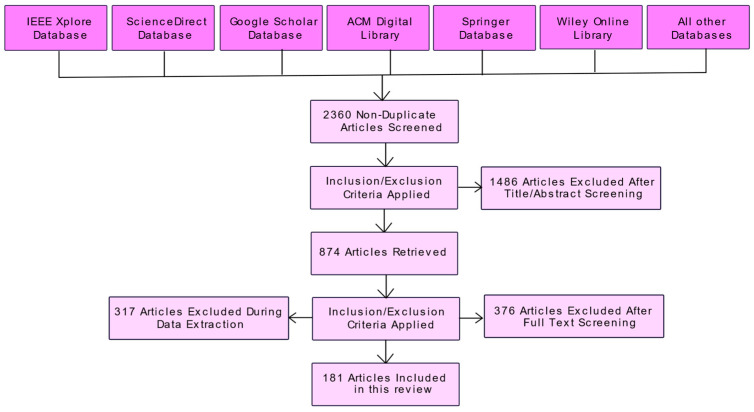
PRISMA flow diagram for the selection process of the research articles used in this review.

Initially, from 2360 articles, duplicates found were removed and, after reviewing the abstracts of these papers, 874 of them were selected for a full-text review. This study included both journal and conference articles. After reviewing the full-text of these papers, 693 papers were excluded, as they used duplicate methods or were published earlier. Finally, 181 papers were studied in this research. Figure 2 illustrates the selection procedure of the articles for this study using a PRISMA diagram.

**Figure 3:**
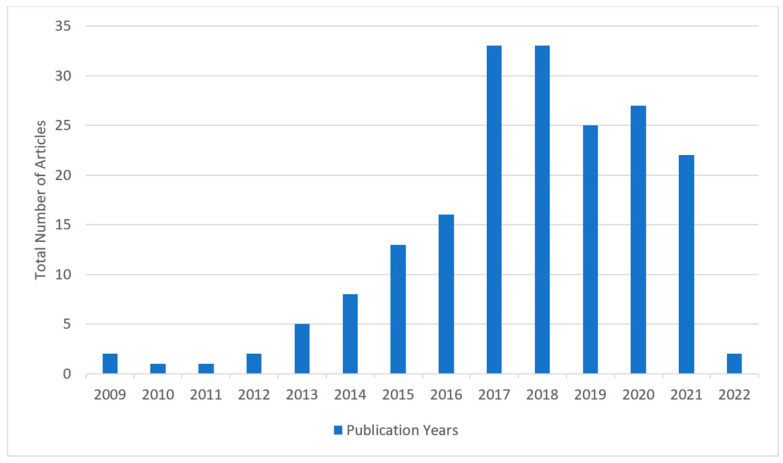
Number and year of publications studied in this review.

The 181 articles studied in this research from 2009 to 2020 are depicted in Figure 3.

The review/survey papers analyzed in this study is elucidated in Table 2.

## 3. Evolving Computing Paradigms and Related Concepts

### 3.1. Cloud Computing

Cloud computing pertains to extending applications via the internet as services, as well as the software and technology that underpins the data centers furnishing these services [1]. NIST formalizes cloud computing [32] as “a model for enabling ubiquitous, convenient, on-demand network access to a shared pool of configurable computing resources (e.g., networks, servers, storage, applications, and services) which can be rapidly provisioned and released with minimal management effort or service provider interaction”. The essential characteristics of the cloud model include on-demand self-service, broad network access, resource pooling, rapid elasticity, and measured service. A cloud infrastructure encompasses software and hardware, extending vital features of the cloud model.

**Figure 4:**
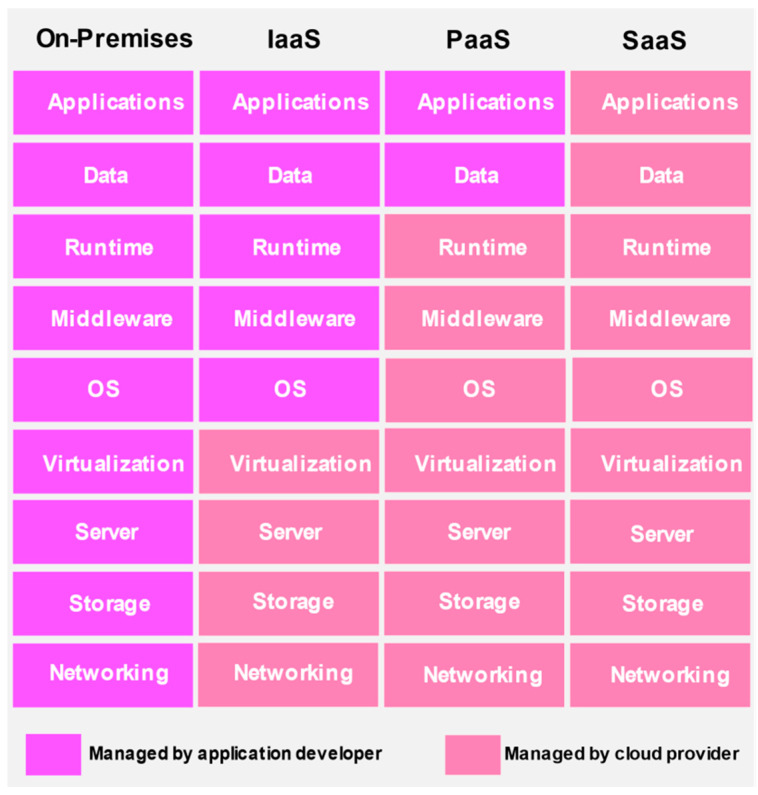
Common cloud service models and their classifications.

The cloud solutions are procurable through the following service models [33]:

- Software as a service (SaaS)—the cloud provider presents consumers with applications accessible via the program interface or web browser, and the consumer has limited control over user-specific applications.
- Platform as a service (PaaS)—On the cloud infrastructure, the consumers are allowed to build and distribute applications. They can exercise control on applications deployed but not on the cloud infrastructure.
- Infrastructure as a service (IaaS)—The customer is furnished with essential computing resources vital to processing, storing, and networking. The user exercises control upon storage, applications, and operating systems, but not cloud infrastructure. The cloud service models and deployment models are depicted in Figure 4.

The cloud solutions are deployable as [33]:

- Private cloud—a particular organization that can also be a third party exclusively owns and controls the private cloud, which can be available on or off-premises.
- Community cloud—the specific community comprises of organizations that share common concerns may provide cloud infrastructure for exclusive usage available on or off-premises, managed by organizations within the community or by a third party.
- Public cloud—the public cloud available on the cloud provider’s premises can be owned or managed by any enterprise and is open to the use of the general public.
- Hybrid cloud—it may be composed of two or more cloud models (private, community, or public); although they are distinct entities, they are bound by technology permitting the portability of application as well as data.

### 3.2. Internet-of-Things

Kevin Ashton, co-creator and executive director of the Auto-ID Center at the Massachusetts Institute of Technology (MIT), in 1999 introduced the phrase “Internet of Things” [34]. The Internet of Things (IoT) [35] characterizes an extensive environment connecting heterogeneous physical objects to the internet to fine-tune the efficiency of real-time ubiquitous applications. As per the International Telecommunication Union (ITU), the Internet of Things (IoT) is a universal framework that connects things which may be physical as well as virtual, distinguished, and incorporated within communication networks, depending on prevailing and emerging collaborative information and communication technologies (ICT) to facilitate enhanced services [36].

#### 3.2.1. Essential Features

The fundamental characteristics of the Internet of Things include [37,38,39,40]:

- Interconnectivity—the IoT may be connected to global communication infrastructure.
- Things-related services—IoT is adept at offering physical/virtual things, privacy, as well as semantic consistency services within the limits of things.
- Heterogeneity—the IoT devices pertain to diverse hardware platforms and networks.
- Constrained resources—the IoT devices encounter computational and energy restrictions.
- Dynamic change—the state of devices and the related environment are subject to dynamic change.
- Uncontrolled environment—the IoT devices are deployed in an uncontrolled setting.
- Massive scale—the devices to be monitored and those that connect with one another are enormous and will continue to surge exponentially into the future.

#### 3.2.2. New Challenges in IoT

As billions of devices are connected globally, data expands exponentially and accumulates 24/7, driving big data to become the current buzzword [41]. The International Data Corporation (IDC) estimates that by 2025, IoT devices may reach 41.6 billion and create 79.4 zettabytes of data [42]. The five Vs of big data [43], namely, volume, variety, velocity, veracity, and value, pose distinct challenges. In the current scenario, the majority of data resulting from IoT devices are managed by the cloud. The resulting cloud IoT synergy poses demands that cannot be tackled by the existing cloud computing model alone [44]. A concise outline of challenges that IoT encounters [13,45,46,47,48] drives the need for edge and fog computing as a solution to manage demands [36] and is outlined as follows:

- Low latency—IoT applications [44] and industrial control systems [49] demand low latency within a few milliseconds that can hardly be met by the existing cloud model.
- High network bandwidth—The escalating amount of IoT devices produce sizable data [50], which may be rendered useless due to high bandwidth usage to transfer it to the cloud or denied due to privacy concerns; hence, entails to be dealt with locally.
- Limited resources—numerous IoT-connected devices possess limited resources to interact directly with the cloud, demanding intensive computation and complex protocols.
- IT and OT convergence—In industrial systems, the confluence of information technology (IT) and operational technology (OT) creates new needs. As offline systems may cause business loss or consumer annoyance, contemporary cyber-physical systems demand continual and safe operation. Thus, the upgradation of the system software and hardware causes concern.
- Intermittent connectivity—When IoT devices have intermittent network connectivity, it is difficult to provide uninterrupted cloud services to those devices.
- Geographical distribution—the majority of IoT devices entail services of computing and storage that are dispersed across large geographic areas, and it is highly challenging to position them at a location that meets IoT demands [51].
- Context-awareness—Local contextual data must be accessed and processed by IoT applications (vehicular networks and augmented reality), for which the physical distance between IoT devices and the centralized cloud is a hindrance.
- Security and privacy—The existing cybersecurity solutions prove to be unsatisfactory to manage IoT applications due to the evolving security challenges [52,53].

### 3.3. Mobile Computing

The computation carried out through mobile phones, tablets, or laptops is noted as mobile computing. The mobile devices offer substantial benefits to mobile users but still encounter limitations due to a low processing capability, battery, memory due to their portable size, and operate at on-and-off network connectivity [2]. Along with resource constraints, mobile computing encounters communication latency, demand adaptability of mobile clients, etc. Thus, these drawbacks cause mobile computing to be inept for applications with demands for low latency, robustness, and when huge data generated from mobile devices need to be processed and stored on it. Moreover, the escalation in mobile device utilization increased the data flow, causing network congestion. Data offloading is a viable solution to mitigate the strain on the cellular network [54]. The existing demerits of mobile computing can be overcome by integrating it with cloud computing [55]. The expansion of mobile computing set the stage for and impacted the cloud and fog computing evolution.

### 3.4. Mobile Cloud Computing

NIST states that the cloud computing alliance with IoT devices and mobile devices facilitates data and CPU-intensive applications suitable for the IoT environment [56]. The concept of Mobile Cloud Computing (MCC) is that it combines cloud computing and mobile applications with sophisticated computing modules processed on the cloud [57]. It enables data processing and storage away from mobile devices, benefitting not just smartphones and a wide range of mobile subscribers. Major computational tasks are moved to the cloud with MCC, improving mobile devices’ battery life [2]. With MCC being grounded on the notion of mobile offloading, mobile devices entrust storage as well as processing to remote units to achieve workload mitigation and optimization in terms of energy, cost, and longevity [45].

The mobile devices’ proliferation entails the efficient management of constrained resources with MCC, as it operates on the synergy between cloud computing and mobile computing. It is capable of operating data-intensive mobile applications as it prevails over battery, memory, and computation power restrictions from the user viewpoint. However, with cellular communication, long-distance data transmission to/from a core network results in higher latency, jitter, and network overhead. This can be overcome if the computation, analysis, and filtering of data occurs at the proximity of the data source, facilitating Fog Computing (FC) and MEC [58]. The limited bandwidth, flexibility, and control, along with an unreliable latency, security, and privacy issues are some challenges faced by MCC [28]. The partitioning of mobile applications by adaptive offloading during runtime allows the management of computer-intensive units of the application [59].

### 3.5. Mobile Ad hoc Cloud Computing

Mobile Ad hoc Cloud Computing (MACC) is an edge computing paradigm involving mobile devices that share resources in a dynamic and temporary network facilitated by transport and routing protocols [2,58,60]. It offers a decentralized network [61] with dynamic mobile devices, accommodating devices joining or leaving the network continually. It favors environments that lack uninterrupted internet connectivity. MACC is the most decentralized, as it comprises mobile devices only.

### 3.6. Cloud of Things

The two concepts of cloud computing and IoT have evolved independently through the years in terms of hardware and software. With IoT facing challenges due to the processing, battery, and storage capacity, these issues can be solved by combining cloud computing and IoT [62]. Cloud computing is capable of filling the gaps in IoT regarding computing, networking, and storage capabilities due to the cloud’s virtually unlimited capabilities and resources. It can assist in implementing numerous IoT applications [63,64]. In the Cloud-of-Things (CoT), IoT devices constitute a virtualized cloud structure. Here, computing is performed over the cloud of pooled resources consisting of IoT devices in contrast to mist computing, where computing is carried out on IoT devices [65].

### 3.7. Mist Computing

Mist computing is initiated to include endpoint connected devices at the extreme edge suitable for self-aware autonomic systems in the near future [2,66]. In the IoT-fog-cloud continuum, mist computing is the first computing point to allow computation, storage, and networking across fog to things. Mist computing forms the superset of MACC, as mist, devices, and networking are not restricted to mobile devices and ad hoc, respectively. It allows utilizing the peripheral component (sensors or actuators) capacity to pre-process data before sending them to the fog or cloud layer [67]. Mist computing aids in large-scale IoT systems development and enriches computational efficiency at the edge of the IoT architecture [68].

### 3.8. Edge Computing

Despite the fact that edge computing has been mentioned in the literature before cloud computing, its significance grew dramatically with the introduction of IoT and the ensuing demands. Edge computing places cloud computing’s services close to the end-user, distinguished by a rapid processing and response time [26]. Edge computing denotes technologies that enable computation to be accomplished at the network’s edge, upon downstream data for cloud services, and upstream data for IoT services [69]. It unfolds the cloud’s network by extending the computation, storage, and resources to the edge of the network, close to the data source, with the resolve to accomplish critical needs of real-time servicing, application intelligence, security, and privacy, along with the network’s requirements for low latency and high bandwidth [13,27].

The significant aspects of cloud computing are its ability to grasp the big picture, process vast amounts of data, perform in-depth analyses, process data in non-real-time, and determine business decisions. Being centralized, entire data must be transferred to the cloud with underlying risks of data loss and data leakage, as security and privacy cannot be ensured, and sensitive information is at threat of disclosure [27]. Edge computing is an extension to cloud computing, which considerably minimizes the volume of data transmitted across nodes, lowering transmission costs and the network bandwidth usage. It leads to utilization and computing efficiency along with energy consumption. Edge computing can be more effective in small-scale, intelligent, real-time analyses. It eliminates the risk associated with the network transmission, ensuring data security. If data become compromised, it impacts only local data. The edge computing architecture is federated, wherein edge devices are positioned between the cloud and terminal devices to tender cloud services to the network’s edge [24,70]. As edge computing shifts service provisioning from the cloud to the edge, it favors IoT application demands, enabling IoT devices to be more scalable and energy-efficient [71]. The cloud–edge alliance involves a typical three-layer model distinguished as the terminal (sensors, cameras, and smartphones), edge (base stations, access points, routers, switches, and gateways), and cloud [27]. In terms of device types, communication protocols, and services, edge computing can be implemented in various ways [9,13,26].

### 3.9. Multi-Access Edge Computing (MEC)

The Mobile Edge Computing standard has been constituted by the European Telecommunications Standards Institute (ETSI) [72], which offers cloud computing and IT functionalities within the Radio Access Network (RAN) in the vicinity of mobile subscribers [71]. As of 2017, ETSI renamed “Mobile Edge Computing” to “Multi-Access Edge Computing” due to increasing interest in MEC by non-cellular operators [73,74]. MEC is a continuation of mobile computing via edge computing, delivering computation and storage to energy and resource-constrained mobile devices [2].

**Figure 5:**
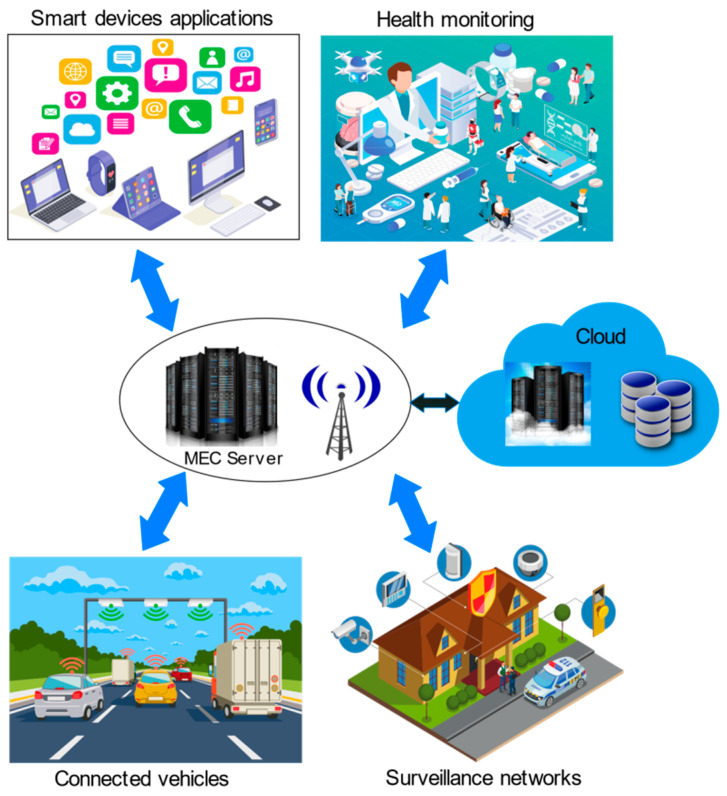
Multi-access edge computing systems—a general architecture.

With Multi-Access Edge Computing, a cloud server is deployed at the cellular network’s base stations. It executes tasks such as enhancing the application’s performance and minimizing network congestion, bandwidth use, and latency for subscribers that cannot be achieved with a conventional network architecture [75,76,77]. Even though the processing and memory capabilities of mobile devices improve, they are still not sufficient to handle compute-intensive tasks, which has led to MCC and MEC models [78]. The MEC architecture is portrayed in Figure 5. MEC considerably reduces the process duration and energy demands of mobile devices by setting up computational and other resources close to the base stations [79]. As base stations serve as crucial access points to several IoT devices, end devices could be directly serviced with just one hop through MEC [80]. MEC showcases a low latency, proximity to the user, location awareness, and geographical distribution. However, it restraints the need to install a dedicated MEC server for MEC services. With the rise in the demand for resources over time, scaling is another major challenge [81]. MEC exhibits the reliability, energy efficiency, and a low latency suitable to diverse applications [82] and outperforms MCC comparably [83].

### 3.10. Cloudlet

A cloudlet has been envisioned by researchers at Carnegie Mellon University, which is a small cluster or data centers capable of computation and storage, positioned close to mobile devices [84,85]. Cloudlet computing shares MCC and MEC particularities, further contending with the demerits of MCC. A cloudlet may be referred to as a mini-cloud [86], offering a secured cloud infrastructure for computing and delivering results to mobile devices and works alongside the cloud. It is positioned at the network edge and is accessible to adjacent mobile units [87,88]. The notion is computational offloading to the virtual machine (VM)-based cloudlets at the network edge from mobile devices [89].

**Figure 6:**
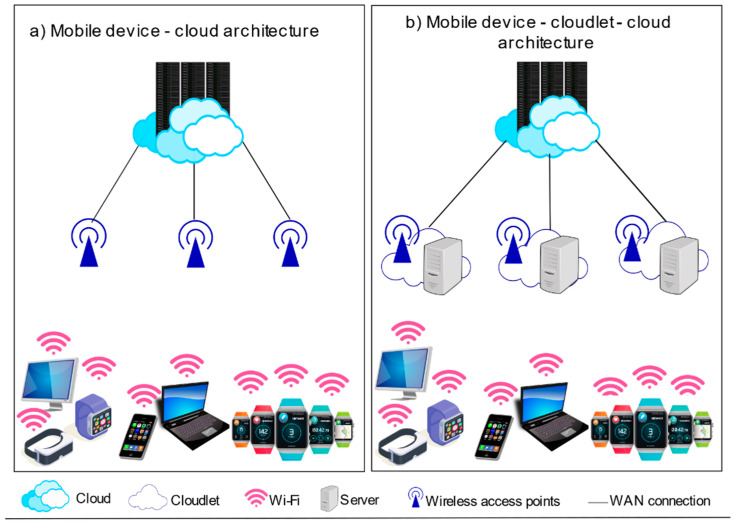
Mobile device–cloudlet–cloud model.

The cloudlet is featured as the middle layer in the three-tier architecture comprising of mobile devices, the cloudlet, and the cloud [84,85] shown in Figure 6. It further possesses connectivity, security, virtualization features, and closeness to mobile users enabling a low latency. Cloudlets are similar to mobile clouds and nearer to mobile devices befitting real-time scenarios. They focus on servicing time-sensitive applications operating under restricted bandwidth conditions and interactive mobile applications with a large resource demand and offer resources at a minimum latency [83]. Cloudlets back mobile clients’ local services by splitting tasks within cloudlet nodes that are nearer to mobile devices. While the cloudlet suits the mobile–cloudlet–cloud structure [90], fog computing is an alternative, supporting huge data traffic with resources located at anyplace within the thing-to-cloud continuum [2]. Cloudlets are alluded to as micro data centers (MDC) at times [91], mirroring conventional data centers of cloud computing. The MDC may be a cloudlet or edge node implemented in between IoT devices and the cloud [2].

### 3.11. Cloud Robotics

Robotics engineering is becoming a vital part of everyday life, with diverse sensors generating big data demanding complex computations [92]. Cloud robotics is a branch of IoT that evolved from the fusion of cloud computing and networked robots [93]. The massive storage capacity of a centralized cloud and broad library of skills can be leveraged by robots to learn with experience. The cloud robotics architecture comprises two levels: machine-to-machine and machine-to-cloud. At the machine-to-machine level, robots determine decisions through a wireless collaboration. The machine-to-cloud level offers a shared pool of storage and computation resources to be allocated as per demands. Cloud robotics capitalizes on the elasticity feature of cloud computing besides others. Additionally, robots-as-a-service (RaaS) stems from considering robots as resources, providing resource sharing services to other robots [94,95,96,97]. The sharing of resources and data between robots through the cloud is integral to CR, along with robots themselves being shared as resources. These systems require standards for enabling coherent, semantic, data sharing, and service provisioning among the robots [93]. Cloud computing delegates fast and robust processing and storage capabilities to robots, along with collaborative learning capabilities through the sharing of knowledge. Recent advancement in this field have incited cloud robotics architecture development and its application in several domains [98].

### 3.12. Fog Computing

The idea of processing at the edge has been around since the 2000s [99,100]. A related concept of cloudlets has been presented in 2009 [101]. The phrase ‘Fog Computing’ has been propounded by Cisco researchers in 2012 [42,102]. Fog computing and cloudlets are related concepts operating at the edge level, with cloudlets deployed at mobile networks and fog computing dealing with connected things [103].

Fog computing is frequently deliberated as a form of edge computing [7,8,104]. Fog computing literally delivers distributed processing, networking, and storage potential nearer to the user [105]. Fog computing is more than a mere deployment of the edge computing concept; it is the pinnacle of reinforcing edge computing concepts [9]. It is not an extension or replacement of the Cloud; instead, it is a new paradigm operating in between IoT and the cloud, with the intent of supporting and enhancing Interaction and integration of the cloud, edge, and IoT.

**Figure 7:**
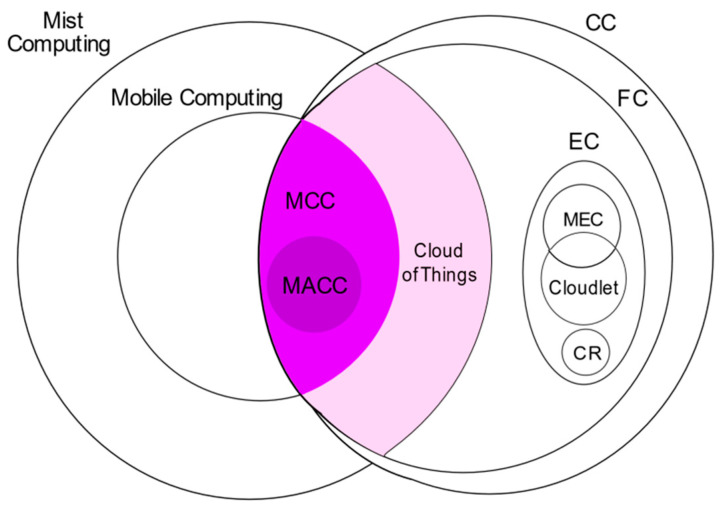
Fog computing and its related computing paradigms.

Cisco describes fog computing [44] as a highly virtualized setup that caters to services of computing, storage, and networking between conventional cloud data centers and end devices, generally but not solely deployed at the network’s edge. According to the OpenFog Consortium [10], fog computing is delineated as “a system-level horizontal architecture that distributes resources and services of computing, storage, control and networking anywhere along the continuum from Cloud to Things, thereby accelerating the velocity of decision-making”. Fog computing adopts a distributed tactic originating from the edge computing model to outdo the limitations of the centralized cloud computing approach [25], with fog nodes positioned anyplace between cloud and end devices. The computing paradigms associated with fog computing are depicted in Figure 7.

#### 3.12.1. Essential Features

The significant trait of fog computing is that the computation, communication, and storage tasks are accomplished close to end-users by capitalizing on the key attribute of fog’s proximity to the edge. The additional characteristics of fog computing [15,106] are outlined as follows:

- Low latency: The proximity of fog nodes to end devices that generate data, such as sensors and actuators, entails a significantly faster reaction and analysis than from the centralized cloud data center. This feature considerably minimizes the data transfer across the internet and enables a low latency to manage real-time applications, notably sensitive to latency and time.
- Save bandwidth: As the fog model allows data computation and storage between conventional cloud and end nodes, less complex pre-processing tasks are handled locally. It dramatically minimizes data transfer across the internet, with endpoints offering fast and premium localized computer and storage services. Transferring only appropriate data to the cloud substantially reduces the network transmission and bandwidth usage, befitting the big data era.
- Multi-tenancy: On account of the vastly virtualized and distributed infrastructure, multi-tenancy in a constrained environment is possible.
- Support for mobility: Due to the direct interaction between fog applications and mobile devices, more control over mobile devices is maintained. Thus, the fog model facilitates better control of users along with mobile devices to administrators and satisfies mobility demands that are location-based and the way information is accessed, resulting in enhanced system performance and service quality.
- Interaction in real-time: Contrary to the cloud, fog applications deliver services in real-time because of their low-latency feature.
- Context-awareness: the nodes and end devices in the fog setting are aware of the contextual location.
- Geographically wide distribution: The fog model’s decentralized architecture facilitates geographically distributed deployment with a large number of widely dispersed nodes. It imparts closer data analysis, rapid big data processing, improved decision-making potential in real-time and location-based services to consumers.
- Wireless access networking: Though fog is deployed in wired environments, it is also suitable for IoT wireless networks.
- Support for heterogeneity: The fog infrastructure encompasses high-speed lines to the data center and wireless access methods to the edge devices. Fog nodes are available physically or virtually, and service interfaces are incredibly dynamic, operating in wired and wireless settings coming from different hardware and software vendors and heterogeneous to cater to the low latency demand of globally distributed applications.
- Seamless interoperability and federation: Owing to its heterogeneous nature, fog nodes and devices originate from various vendors and are generally deployed in diverse settings. For the effective interaction of devices from different providers, fog computing must enable interoperability with federated services across domains. Thus, to allow interoperability and cooperation across diverse resources and devices, fog computing employs policies for resource management.
- Real-time analytics: With data collected and processed close to the sources, real-time analytics is possible.
- Scalability: Fog computing exhibits scalability and adaptability to varying conditions with the data load, resource pooling, network demands, and flexible computing.
- Support for industrial applications: As computing and analyses are conducted in real-time, industrial applications widely benefit.
- Security and privacy: Fog computing brings facilities nearer to end consumers while ensuring privacy and security of sensitive and private data using integrity checking, access control, and encryption methods by fog nodes. Moreover, it can mitigate the vulnerabilities associated with system upgrades and limit updates at the fog end.
- Low energy consumption: as fog nodes are spatially distributed and do not require a cooling system, fog computing is more ecologically friendly; communication within a short-range as well as energy management rules obviously minimize the communication energy use.

In addition, fog nodes are expected to possess features such as autonomy, heterogeneity, hierarchical clustering, manageability, and programmability to fog implementation.

#### 3.12.2. Architecture

The illustration of the fog architectural model has recently been a prominent area of research. Extensive related research alluded to the architecture comprising of three-layer [15,17,107,108,109]. Moreover, the N-Tier architecture recommended by the OpenFog Consortium may be regarded as an enhancement of this three-layer model [10].

1. Three-Layer Architecture

**Figure 8:**
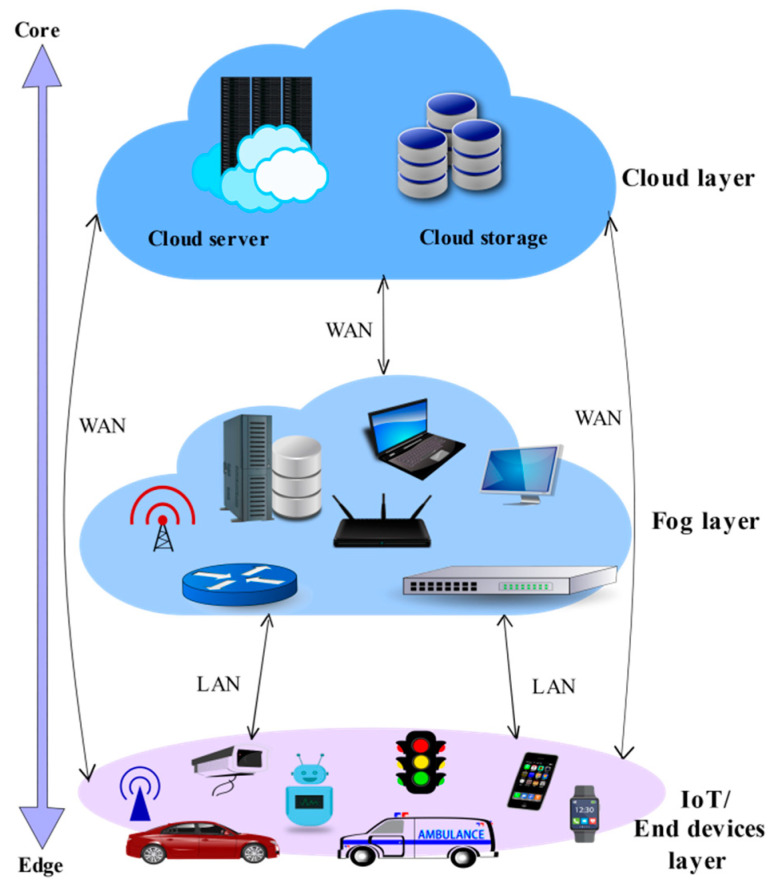
Typical cloud–fog computing architecture.

The basic three-layer model portrayed in Figure 8 stems from the fog computing concept being an essential extension to the cloud computing model, with the fog layer posing as an intermediate layer between the cloud and IoT devices [15].

  - IoT Layer

It is nearest to the end-user’s physical setting. Sensors, smart cars, drones, smartphones, tablets, and other devices compose this layer. Even though some of these devices possess computational capabilities, they are used as mere smart sensing devices at this layer. Overall, these devices are widely distributed geographically to sense and transfer data to the next higher layer for the sake of storage and computation.

  b. Fog Layer

This layer comprises numerous fog nodes and forms the basis for the fog computing architecture. As per the OpenFog Consortium [10], fog nodes may be a physical or logical network element that enforces fog services. Thus, fog nodes have a direct connection to extend services to end devices. On the other end, fog nodes are linked to the cloud infrastructure to deliver and receive its services and benefits.

  c. Cloud Layer

The centralized cloud infrastructure composes the majority of this tier. It comprises several servers with advanced computational and storage capabilities offering a variety of services. Dissimilar to typical cloud computing architectures, the fog model can ease the burden on cloud resources by efficiently transferring computational services from the cloud layer to fog and enhancing productivity.

2. OpenFog N-Tier Architecture

**Figure 9:**
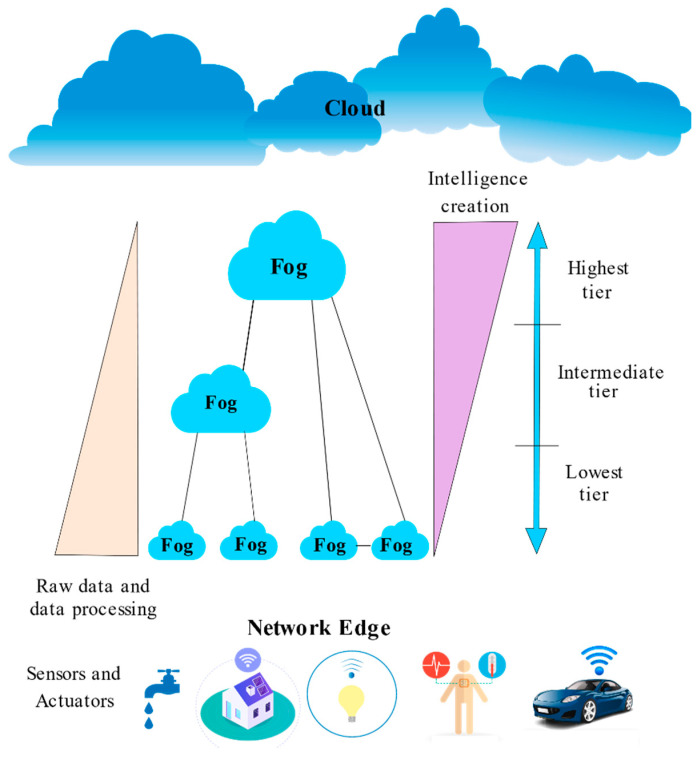
N-tier architecture.

The OpenFog Consortium’s recommended N-tier architecture [10] is rendered in Figure 9. Its primary intent is to offer a standard guideline for implementing fog computing in a given circumstance. Though fog systems are deployed in a scenario-specific manner, the core elements of the architecture are apparent to every fog deployment. Endpoints (or things), fog nodes, and the cloud are the three main components of the idea. In addition, multiple layers of fog nodes (N-tiers) may constitute the fog layer; when the nodes are more distant from the end devices, improving computing potential and intelligence are acquired.

The higher levels of the fog layer refine and collect more pertinent data; therefore, enhancing intelligence. The scenario-specific needs ascertain the number of tiers in a particular implementation. Furthermore, fog nodes connected in a mesh on each layer are adept at providing added characteristics such as fault tolerance, elasticity, load balancing, etc. Thus, the fog nodes may interact both vertically and horizontally.

Fog nodes may be categorized based on their closeness to the cloud and endpoints:

- Lowest tier: with the primary focus on the acquisition, normalization, and collection of data obtained at the sensors, and the actuators are managed by fog nodes.
- Intermediate tier: filtering, compressing, and altering data received from the bottom layer is the responsibility of fog nodes in the intermediate tier; on average, these nodes are better at analyzing data.
- Highest tier: aggregating data and eliciting knowledge from it is the intent of fog nodes at this tier.

3. Seven-Layer Architecture

**Figure 10:**
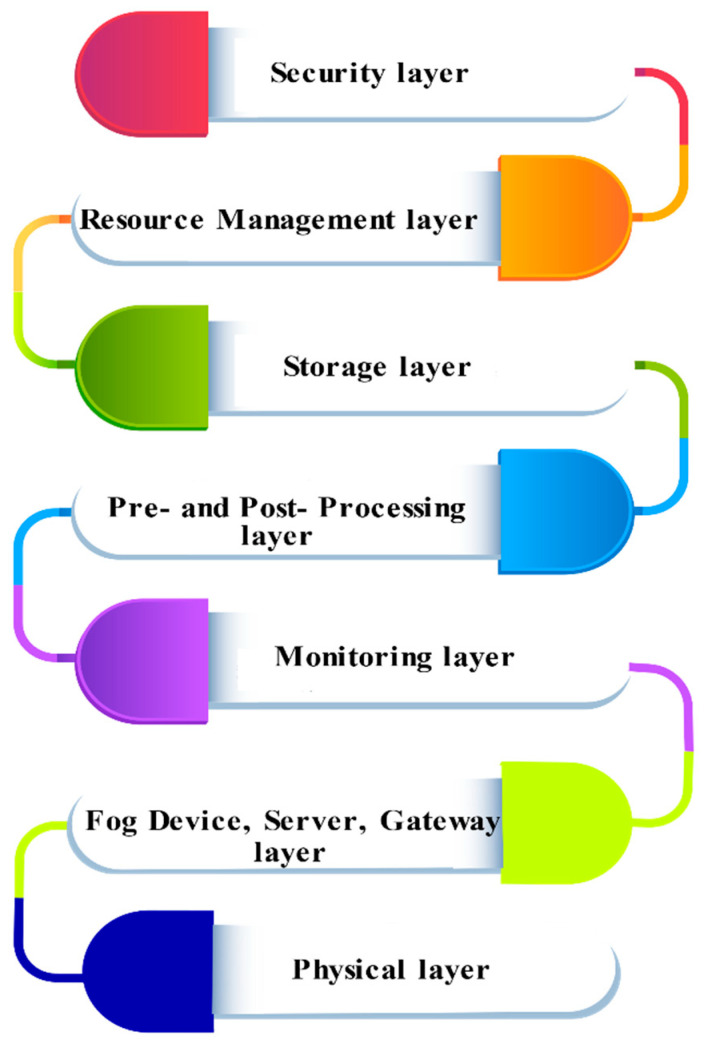
Typical fog computing architectural layers.

A fog computing model positioned between the cloud layer, and edge devices extend services of processing, network, and storage to IoT devices, with the primary intent of minimizing latency for time-critical applications. The services offered by the fog model are limited compared to the sophisticated cloud data centers. In line with various fog architectures presented by researchers [110,111] with diverse layers, a reference architecture comprising distinct layers with designated tasks is featured here and depicted in Figure 10.

  - Physical layer

The sensors are the devices that serve as the primary data source producing diverse data in a fog setting. The data may be from smart devices and homes, autonomous vehicles, closed-circuit television (CCTV) monitoring, traffic systems, sensors tracking temperature and humidity, etc. Alongside physical sensors, the physical layer also comprises virtual sensors, which also produce data as well.

  b. Fog device, server, and gateway layer

An individual device or IoT could be a fog device, server, or gateway. The fog server entails configuration, computation, and storage capabilities, higher comparably in order to handle the fog device and gateway. It further pertains to hardware configuration, devices it can handle, network connectivity, etc., with its role defining it to be distinct or an IoT fragment. A set of virtual and physical sensors are attached to the fog device. In the same manner, a set of fog devices could be attached to a fog server. A specific group of fog devices connected to a particular fog server can interact as and when required. The processing has to be performed at multiple fog servers and devices to determine a proper decision. The level of fog devices and servers is in charge of handling and servicing data on storage and hardware configuration and connectivity for the fog servers and devices. The processing demands of different applications are addressed at this layer.

  c. Monitoring layer

The system operation, services, resources, and responses are tracked by the monitoring layer, which facilitates identifying appropriate resources in the midst of the operation. If a possibility arises for the fog device as well as the fog server where resource availability becomes negative for processing or storage, assistance from peers may be sought. The system monitoring unit aids in efficient decision making in such unforeseen scenarios and resource failure by tracking the present resource consumption, usage and then estimating resource demands into the future. The performance prediction component tracks and forecasts the performance of the fog system depending on the resource availability and system load. This unit is necessary to keep up with the relevant Quality of Service (QoS) demands in SLA (service level agreements). The occurrence of repeated SLA violations may increase system costs due to penalties for the provider. Even though this issue cannot be completely ruled out, SLA violations can be greatly reduced if the performance prediction component foresees the system’s performance and usage.

  d. Pre- and post-processing layer

The multiple components of this layer are distinctly concerned with the data analysis at the basic as well as advanced levels. The data accumulated are subjected to analysis and filtering with trimming alongside a reconstruction performed when needed. Once data processing is accomplished, the component called data flow finalizes the process if the data have to be stored locally or in the cloud. Fog computing insists on stream processing, which processes and stores minimum relevant data at the edge, as all data generated may not be useful. As per the application, requisite data trimming can be performed where the mean value of the data within a minute or hour could be stored if the sensor produces data every second. In instances where data values do not differ significantly over time but tend to affect performance, the number of readings taken can be reduced. Though perfect accuracy would not be achievable, application requisites may be attained. The data reconstruction module reconstructs the data as per the pattern in which data is generated in times of incomplete and faulty data produced by sensors to avoid application failure or an interruption.

  e. Storage layer

The storage module is accountable for data storage by storage virtualization. The unit referred to as the data backup affirms data availability and minimizes data loss caused by system failure by creating a backup of critical data. It also periodically customizes schemes of data backups. By storage virtualization, a collection of storage devices functions as a single device, enabling manageability and maintainability; thus, offering an enterprise-level operation, at low-cost hardware and storage.

  f. Resource management layer

The resource management layer addresses resource allocation, resource scheduling, and energy saving. The reliability unit at this layer ensures the application of scheduling reliability, system reliability, and the allocation of resources. Maintaining reliability is critical, as a complex fog system encompasses IoT and fog devices alongside the cloud with many failure possibilities. The scalability component assures that the fog resources are scalable when resource demand surges at peak hours. The fog model aspires to offer horizontal as well as vertical scalability, while the cloud platform ensures horizontal scalability. With distributed resources for processing, networking, and storage, the resource allocation unit allocates, deallocates, and reallocates resources. As multiple applications are run concurrently in fog systems, the scheduling of applications is managed by the application scheduling component. The energy-efficient resource management is handled by an energy-saving component at this layer, which reduces operational costs.

  g. Security layer

The security layer deals with all issues that relate to security, such as encrypting communication, securing stored data, preserving fog users’ privacy, etc. Similar to cloud computing, fog computing is deliberated as a utility model. As users connect to the cloud for availing services, users also connect to the fog system for services; however, the fog middleware manages interactions with the cloud. The provider must authorize the user attempting the service connection, and the authentication unit authenticates the user’s request to avail fog service. To ensure security and evade security breaches by intruders, every interaction has to be encrypted. The component of encryption encrypts the connection between IoT devices and the cloud. The majority of fog components is connected wirelessly, and ensures security is critical. Fog systems acting on users’ private data should not reveal those without proper user approval. At times where users accept a provider’s security policy without reading it, it is critical to assure that user privacy is upheld.

  h. Application Layer

Despite the fact that fog computing emerged to handle IoT, various applications pertaining to the Wireless Sensor Network (WSN) back fog computing. The majority of latency-sensitive applications leverage fog’s utility model that delivers a cost-effective and enhanced service quality. The systems deploying the augmented reality (AR) and virtual reality (VR) concepts can harness the fog computing attribute of processing in real-time. Augmented reality adds virtual content into a user’s real-life experience. Virtual reality, on the other hand, produces a computer-generated simulation of a virtual world. With AR and VR reckoned to transform the world in the near future, association with fog will ensure a continued refinement. 

#### 3.12.3. Fog Computing Applications

**Figure 11:**
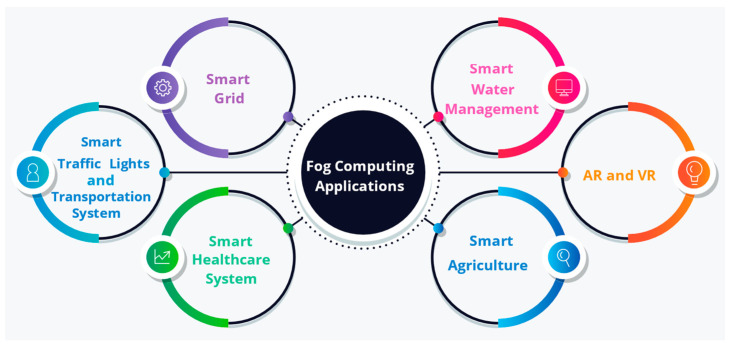
Applications of fog computing.

Numerous applications, such as smart homes, smart cities, smart grids, smart water management, smart transportation, smart agriculture, augmented reality, virtual reality, smart healthcare, and smart vehicles, compel the fog framework’s efficient services. The fog computing applications described here are depicted in Figure 11.

- Smart grid

A smart grid extends the reliable, efficient, automated electricity distribution model aiming to cut down operation costs, enhance transmission efficiency, and offer to smoothly integrate with systems involving renewable energy [112]. It further enables service providers and consumers to track and regulate the real-time price, output, and consumption of power [113]. The fog systems play a key part in favoring smart grids within smart cities, minimizing electricity bills. Here, the data produced by the fog devices can be locally analyzed and filtered by fog collectors deployed at the edge and transmitted to the cloud for complex analysis, visualization, and long-term storage [114]. As per varying demands, such as a low-cost and energy, smart grids allow switching over to any other supplies of energy, such as solar or wind, with edge/fog devices gathering local data to decide in real-time [115].

b. Smart traffic lights and transportation systems

In smart traffic light applications, smart traffic lights aid in lowering traffic congestion, noise, fuel consumption and avert accidents thus, improving the driving experience. These connected lights functioning as fog devices are adept at detecting the ambulance’s flashing lights and changing the traffic signal to open lanes for the ambulance to travel through [116]. It recognizes pedestrians and cyclists and figures out the speed and distance of vehicles approaching and collaborates to provide warning messages to adjacent vehicles. Moreover, smart lighting is switched on when movement is detected and turned off automatically when traffic passes. This system comes handy in averting accidents, maintaining low and steady traffic, and collecting pertinent data to enhance performance. With huge data produced by intelligent transportation systems (ITS), processing using centralized model results in large delays. In this sense, fog nodes at particular intersections could be utilized to analyze data locally and brief people on current situations, substantially lessening the delay [117].

c. Augmented reality (AR) and virtual reality (VR)

Augmented reality overlays digital and virtual content into a physical environment. It is highly time-critical, warranting responses in real-time. Moreover, it is extremely latency-intolerant, as even a minor delay may impact user experience, effectuating a negative response [116]. For this reason, fog computing has the potential to become a key player in the augmented reality domain, as computer-intensive jobs can be offloaded to nearby fog devices. This also holds true for the virtual reality (VR) field, which offers real-world experience through a simulated environment and is generated by computer technology.

d. Smart healthcare system

When distant cloud servers are used to process and store enormous healthcare data generated from sensors, the huge data transmission, defining of location, and access latency pose critical challenges [118,119]. As healthcare datasets increase, there is a higher possibility of error occurrence during processing as well as transmission. Even a minute data analysis error may instigate the administering of an inappropriate treatment that could cost a human life. The patient health data is sensitive; hence, security and privacy preservation are vital. The integration of fog computing into healthcare enhances efficiency and quality as the computer and storage are provided nearer to end devices, which permits aggregation, processing, local storage, and real-time analytics. It further displayed a low latency, mobility support, privacy, and location awareness, and experiments demonstrated an enhanced system response time along with an improvement in energy consumption [120].

e. Smart agriculture

As agriculture caters to the food supply chain, it plays a prime role in smart city schemes [121]. With smart agriculture, sensors installed in field vehicles gather data on plant growth and field climate conditions. Moreover, the field can be sensed from the sky using air balloons. These sensing activities can be effectively accomplished by fog computing, and agricultural lands can be managed and tracked through sensor nodes’ alarm notifications.

f. Smart water management

As far as sustainable smart cities are concerned, smart water management is crucial. It supervises the quantity of water consumed, transported and anticipates the use of water in the future. Above all, it enhances the water system of the city to be more reliable, sustainable, and efficient, as it assists in mitigating water loss using sensors that collect and analyze data of the water system [121].

## 4. Challenges and Opportunities

Despite the fact that cloud computing has been around for a long time, it still confronts problems. Cloud security, privacy, confidentiality, availability [122], and sustainability [123] are among them. The dependability of cloud services is an issue as well; when a limited number of data centers offer critical functions, it might be disastrous if one of the data centers goes down [124]. Cloud data centers require immense energy to operate, which requires mitigating energy usage by resource provision optimization policies. The cloud networking infrastructure faces challenges pertaining to network utilization, data congestion, cloud federation [125], etc. As the IoT devices arrived, an emphasis was placed on reducing energy and resource usage, and critical difficulties included increasing the battery life or optimizing the energy utilization of smart devices [126]. The security of IoT devices and withholding the privacy of sensitive data collected by the connected devices pose unique challenges [127]. The availability, reliability, scalability, and interoperability of IoT networks are labelled to be challenging.

Edge computing, which moves computation to the network’s edge, poses a number of complications, such as focusing on the programmability of edge devices, naming schemes for a large number of edge devices, including security, privacy, data abstraction, service management, and optimization issues [41]. With fog computing still in its developmental stage, it faces many open challenges. It has difficulties similar to edge computing due to its correlations, and the notable challenges include programmability, managing heterogeneous systems, providing security, interoperability, mobility, scalability, federation, and energy/resource efficiency [20,128].

**Figure 12:**
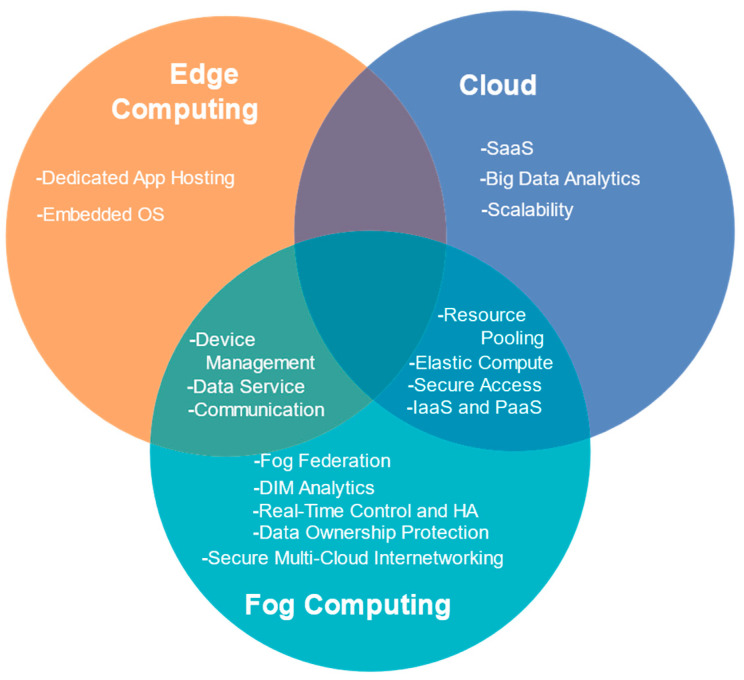
Cloud, fog, and edge computing alliance.

Fog computing is a more generic model compared to related paradigms due to the far-reaching scope and presence in the Thing-to-Cloud continuum. The comparison and features of the fog, edge and cloud [2,129,130,131] are displayed in Table 3 and Table 4. The association between cloud, edge, and fog computing [132] is shown in Figure 12. Fog computing is imminent of offering amelioration in the near future in an open-standards setting of connected devices, apparent when the IEEE Standard adopted the Open-Fog Reference Architecture [133]. Hence, our cynosure for the rest of the paper is on challenges and future research directions pertaining to fog computing.

### 4.1. Fog Computing: Open Challenges

**Figure 13:**
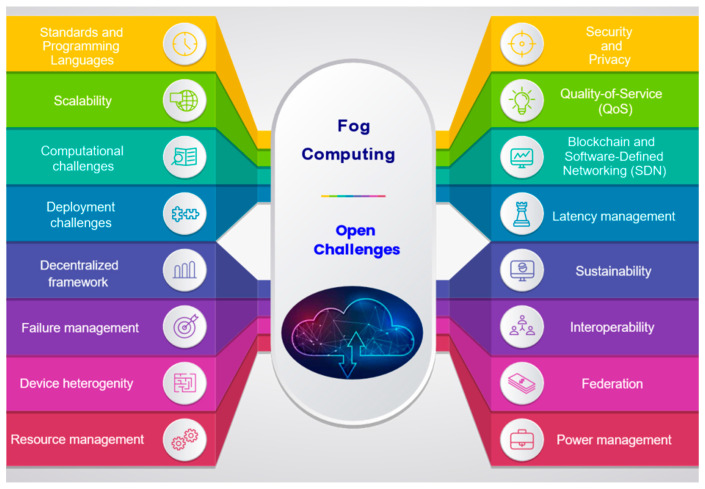
Fog computing—open challenges.

The fog computing paradigm has evolved from the cloud computing utility model. With IoT proliferation, computations closer to the network edge significantly minimize the cost of computing and data offloading at the cloud. However, processing at the edge poses numerous challenges pertaining to devices, security, the network, integrating fog, and IoT, which the distributed fog system has to deal with [28,44,110]. The open challenges identified are pictured in Figure 13.

- Standards and programming languages

The fog structure is distinct from the cloud as it extends cloud services to end-user devices, warranting upgraded standards and associated programming languages, along with effective user interfaces and network protocols for IoT device management.

- Scalability

Scalability is a key issue for systems involving extensive IoT applications on fog, and exploring optimal algorithms that illustrate the fog system’s complexity would be valuable. In the fog model, time-critical tasks are executed at the fog, and others are moved for processing to the cloud. Ascertaining when fog resources are utilized optimally depending on service type, user count, and resource availability are significant.

- Computational challenges

The Fog system continually interacts with the cloud servers. It intends to respond to users within a stipulated duration and forward complex computer-intensive tasks to the cloud, which may take longer. The parts of computation that are unrestricted by response time are sent to the cloud, while others are carried out at the edge for a minimum computational cost. The challenge lies with figuring out which computer tasks are to be executed at the edge and offloaded to the cloud.

- Deployment challenges

The fog system has to be precisely deployed to subdue latency. Factors such as the type and task amount performed at a particular tier, fog device capability, and reliability, and the number of sensors determine implementation decisions. As per the application requirements, resource scaling, as well as shrinking, are carried out without hindering the operation of ongoing services. OpenFog recommends the N-tier fog model from s mobilization viewpoint; however, escalating the fog layer levels may instigate delays, which require defining the number of levels for the specific application.

- Decentralized framework and failure management

The decentralized fog entails a high likeliness of fog device malfunction relating to the software, hardware, power source, mobility, as well as connectivity issues considering an unreliable wireless connection, linking the majority of fog devices. The fog system is adaptable to a minor disruption and resource shortage. The fog node failure may make its respective virtualized resources unavailable, and related issues, such as latency and migration, have to be dealt with for resource availability at downtime. The decentralized fog results in the repetition of code at edge devices, and this redundancy has to be checked. The random distribution of network resources at the edge complicates connectivity, which can be rectified by deploying a middleware that manages resources to the demanding application. The small client services are disseminated from the cloud to the edge, and acquiring such services from fog systems is quite challenging. The fog system manages billions of IoT devices; hence, provisioning services to all fog devices is arduous. The portability of the fog’s edge node requisites ubiquitous fog computing. With fog being distributed, the preciseness of computation needs to be confirmed as its applications demand consistency.

- Device heterogeneity and resource management

Fog computing sets the stage for numerous heterogeneous technologies to offer IoT services with a key challenge of linking resources from diverse platforms. It is vital to examine algorithms that are competent at handling scheduling, synchronizing for the effective utilization of IoT devices that are short on resources. The diversified nature of edge devices has to be emphasized by the fog architecture at the device as well as the network levels. Utilizing heterogeneous devices in a diverse fog setting with varying application demands is strenuous. Numerous IoT devices from diverse hardware and software vendors add to the complexity factor. When the edge lacks computational resources, it can be acquired and assigned from among the fog nodes setting up a common pool of computing, network, and storage resources, availed by applications as per demand. The heterogeneity of fog devices and resources in the dynamic fog setting enables resource scheduling and allocation to be more challenging than that of the cloud, with utilizing idle resources being fog’s top priority.

- Security and privacy

The heterogeneity of devices makes the fog framework vulnerable to various attacks due to its deployment in a not-so-secure setting. As fog nodes are positioned between the cloud and end-users, fog computing is susceptible to security issues. Assuring the privacy of sensitive data originating from sensors is critical. The fog-based Distributed Denial of Service (DDoS) attack is highly destructive, as diverse malignant devices overwhelm resource-limited end devices with fake service requests. Another such attack is the Man-in-the-Middle Attack (MMA), which discloses sensitive private data. The physical components of IoT devices can also be attacked, referred to as a physical attack based on the protection level and implemented location.

- QoS

The fog framework encompasses devices from the cloud to the edge, and the fog nodes are to provide end-to-end services adhering to users’ service-specific QoS features. The fog system is entitled to manage the distribution of computing and storage to the cloud while orchestrating heterogeneous edge devices. Hence, it is necessary to dynamically integrate cloud servers and fog devices.

- Blockchain and Software-Defined Networking (SDN)

In fog-based IoT settings, blockchain technology can provide a secure framework for controlling data and information exchanges amongst independently operating devices. To improve privacy and security, blockchain offers the safe transmission and storage of digitally signed documents. As a result, an additional study into this technology is critical in order to offer and improve methods for securely transmitting data between IoT devices, utilizing a trustworthy approach such as a time-stamped contractual handshake.

Furthermore, Software Defined Networking (SDN) is a networking technology that may be used in conjunction with fog technology to enable effective data exchange and resource collaboration. SDN may also bring intelligence to fog-based IoT networks, among other things. SDN may also be utilized to protect fog-based IoT infrastructures. The authors, for example, developed a hybrid network design for smart cities that included SDN with blockchain. As a result, research into SDN and its integration with blockchain would be helpful in providing an efficient architecture for sustainable smart cities.

- Latency management

Latency control is required in fog computing to guarantee an acceptable level of Quality of Service. As a result, research into various latency management techniques would aid in delivering services with the least amount of delay and ensuring a higher QoS throughout the system. The estimate of resources is another key topic in fog computing. It aids in allocating computing resources depending on various policies, allowing for the correct allocation of resources for future computation. In order to attain the necessary QoS, a comprehensive study into various resource estimate policies in terms of multiple aspects such as user attributes and experienced Quality of Experience (QoE) would be useful.

- Sustainability

In order to reduce the total carbon footprint, sustainability which refers to the utilization of renewable energy supplies, energy harvesting, and energy-efficient architecture, is a crucial necessity when building fog-based IoT architectures for smart cities. Dense IoT end-devices and fog computing servers are predicted in smart cities. As a result, the smart city infrastructure would suffer considerable energy constraints. As a result, it is critical to research various methods for increasing the energy efficiency of fog-based IoT systems without sacrificing QoS, which could be accomplished through energy-efficient caching methods.

- Interoperability and federation of fog

Another essential prerequisite for accomplishing the goal of a fog-based IoT and sustainable smart cities becoming a reality is interoperability. Because of the large number of heterogeneous IoT devices running on multiple protocols, the interoperability of fog-based IoT systems in sustainable smart cities is difficult. The fog-based IoT architecture should provide interoperability so that various systems and devices can correctly comprehend and use each other’s functionalities. On that account, intense research efforts are recommended to create frameworks that allow interoperability for fog-based IoT systems in sustainable smart cities.

On the fog, requests are processed at proximity, mitigating latency. If numerous latency-sensitive applications were to request services, the interoperability of the Fog clusters and its servers along with federation would be required so that a fog device can request its peers to manage processing to avoid cloud involvement that increases latency.

- Power management

Fog nodes manage innumerable end devices, as in sensors, and when fog nodes are employed as needed, they substantially multiply active nodes, increasing the whole system’s power consumption. Hence, power has to be managed effectively in large fog systems. One such option to study would be integrating the fog nodes in specific applications and moving tasks among nodes. The majority of fog devices are power-constrained, and efficient energy utilization is essential.

Table 5 furnishes the summary of open issues and potential solutions concerned with fog computing.

### 4.2. Future Prospects of Fog/Edge Computing

**Figure 14:**
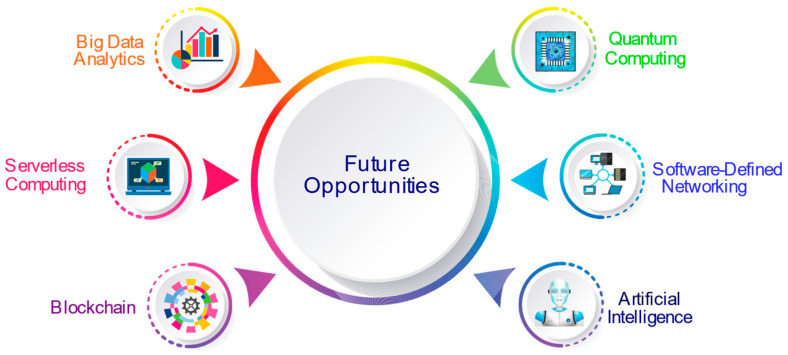
Future opportunities—fog computing and other evolving computing paradigms.

The technological possibilities that may lead fog/edge computing paradigms into the future are portrayed in Figure 14 and detailed as follows:

#### 4.2.1. Big Data Analytics

The proliferation of the ubiquitous IoT has led up to an overwhelmingly immense amount of data generation, inferred as big data [134]. Big data entails ever-expanding datasets, which are heterogeneous in nature, comprising of structured, semi-structured, and unstructured data [135]. It garners potential for opportunities as well as challenges, including the five Vs [136]. Thus, big data analytics is a promising solution that processes the humongous big data and transforms it into smart data, imparting actionable insights into making data-driven decisions [137]. The key feature of fog computing and edge computing models is the potential to quickly store and process data, benefiting real-time applications and playing a crucial part in efficient business operations [138,139].

#### 4.2.2. Serverless Computing

Serverless computing facilitates an easy and hastier IoT application development by eliminating the need to manage a real infrastructure [140]. It is also referred to as the Function-as-a-Service (FaaS), implementing code as independent functions through dynamic resource provisioning, which enhances the runtime infrastructure scalability [141,142]. Integrating serverless computing to the edge computing model increases the computation speed of data generated and processed by IoT applications deployed on edge devices [143]. As individual functions are executed on edge devices, the response time, latency, and energy consumed is decreased, and the reliability is improved.

#### 4.2.3. Blockchain

Blockchain is a novel concept to store data as a chain of blocks to enhance data security [144]. It is a super-secure method to store, authenticate, and protect data, which promotes trusted transactions. Blockchain usually revolves around securing cryptocurrency with real potential being transparent and immutable. It utilizes the distributed ledger model to secure transactions and is decentralized in nature, providing accurate and efficient transactions, evading intermediaries. Blockchain is engaged in offering services pertaining to finance, voting, supply chain monitoring, and smart contracts. It can be deployed to secure data generated by IoT applications [145,146].

#### 4.2.4. Quantum Computing

The emerging field of quantum computing extends a substantial computational lead over classical computing by leveraging the quantum physics principles of entanglement and superposition [147]. With unimaginably swift quantum computers, calculations are performed and stored using quantum bits referred to as qubits, which allows number crunching and problem-solving at an exponential scale. Seemingly unsolvable complex tasks, predicting viable solutions to issues, and the processing of a massive amount of data can be handled with absolute ease by quantum computers. They can further enhance computational efficiency, security, and energy efficiency [148]. Quantum computing can be combined with ML and DL techniques to predict the resource demand and handle an efficient resource and energy utilization at fog and edge layers [149,150]. Quantum computing is in its budding stage, with research efforts underway at an accelerating pace [148].

#### 4.2.5. Software-Defined Networking

Software-Defined Networking is an upcoming paradigm that overcomes the vertical integration issue by separating the control logic of the network from the underlying switches and routers, enabling a logical network control centralization [151]. It makes it simpler to manage a flexible and reliable network, introduces new networking abstractions, and leads to network evolution. SDN overcomes conventional network issues by enhancing the virtualization, security, energy efficiency, and network reliability, optimizing the network topology, managing complexity, service orchestration-benefitting fog, and edge computing [152,153].

#### 4.2.6. Artificial Intelligence (AI)

Artificial intelligence is a key field of computer science, where machines mimic human intelligence/behavior and is already transforming the world. The accelerating ability of machines to learn and act smart is gearing up to drive even more businesses and technologies. AI, collectively with its subfields of machine learning and deep learning, help businesses save cost, enrich customer experience, communicate effectively, streamline workflows, and obtain insights for better decisions. ML is the ability of a machine to learn without involving explicit programming. It can analyze huge datasets and offer actionable insights. DL, which is a subset of ML, is capable of handling complex computational tasks. AI has begun to see the light of the day with automation and implementation occurring at a large scale and fast pace. Likewise, intense research efforts are underway for integrating fog and edge computing with artificial intelligence to enhance the overall performance, including resource, energy management, security, and reliability [154,155,156].

## 5. Sustainable/Green Computing in Fog/Edge

Sustainable/green computing is the efficient management of computational, communication, and storage devices through convincing design and manufacturing practices with a reduced impact on the environment [157]. The last decade has seen sustainable/green computing permeating fields of social computing, mobile computing, agent systems based on AI, as well as the Internet of Things. IoT nodes possess power constraints and connecting with the internet makes them vulnerable to attacks. For IoT to be sustainable, energy and security are the two key aspects to be emphasized.

### 5.1. Energy Sustainability

With IoT services pervading all aspects of our lives, energy-constrained IoT devices spark concern while considering sustainability. The massive number IoT sensors and actuators deployed necessitate a continuous and persistent power supply. As the IoT node size reduces, the size of the battery also decreases. In light of the current trend to enhance IoT device functionality, formulating sustainable solutions for confronting power constraints is essential [158].

Numerous research efforts have been oriented towards energy harvesting for self-sufficient IoT functioning, alongside tackling IoT security issues. The energy consumed by digital and smart gadgets has become concerning. Energy harvesting from renewable energy sources can power a myriad of IoT sensors [159,160]. With IoT sensors having a battery that lasts for a limited time, frequent charging or replacement is not viable at all times. Hence, energy harvesting from renewable energy such as kinetic, solar, thermal, etc., seems plausible [161]. Moreover, energy harvesting this issue can be handled by deploying an efficient data transmission policy [162], with almost 80% of a sensor’s energy being depleted on data transmission. Even though efforts for enriching the energy efficiency of IoT systems are underway, they hardly match the proliferating pace of IoT services/dependence [158].

### 5.2. Security Sustainability

IoT sustainability emphasizes the security of data and devices. Securing data involves handling confidentiality and integrity aspects, whereas device security concerns defense against stealth attacks. Energy-harvesting chips are susceptible to malicious attacks, including DoS attacks that disrupt sensors. Both the criteria of energy efficiency and security characterize the IoT sustainability while, at the same time, challenging IoT progress [163] as IoT devices are power constrained, which demands a refined, lightweight energy and security framework.

According to a study, 70% of connected devices are at risk of cyber-attacks [164]. Furthermore, vulnerable smart devices are estimated to cause 25% of all industrial attacks [158]. As IoT devices are resource-constrained, they are highly prone to attack than desktops or laptops. As the battery size decreases, it can hold less energy, which in turn reduces the availability of resources that provide security. Hence, lightweight security mechanisms suitable for power constraint devices are essential, as traditional security solutions designed for resource-rich devices consume more energy, owing to more computations. Research shows that the advanced encryption standard, as well as the elliptic curve cryptography, offer a lightweight cryptographic solution with an evaluation based on resource limitations, chip space, latency, and throughput [165]. For the IoT systems to be sustainable, the balancing of aspects such as energy efficiency, power consumption, performance, and security is required [158].

## 6. Confluence of ML and Fog/Edge

The conventional cloud model falls short of fulfilling IoT application necessities due to the enormous data generated from IoT devices [166]. Transmitting the overwhelming IoT data to the cloud would cause network overhead, consuming bandwidth, and latency issues [167]. Hence, to cut back on the data transfer cost as well as network delays, service providers are steering towards the fog and edge computing [168], with an additional opportunity for enforcing security and privacy [169]. The IoT systems comprise edge equipment, sensors, and actuators with latency, bandwidth, and security necessities [166]. The fog computing technology of extending computer and storage to network’s edge solves processing and networking impediments [167], enabling rapid processing close to the data source [170]. The complexity and dynamism of fog computing with its communication networks facilitating low latency makes sophisticated computation possible in a conducive environment. Fog computing confers societal benefits through its range of applications, namely, healthcare, Industry 4.0, autonomous vehicles, smart cities [171], etc.

Despite that, it encounters performance as well as security setbacks. As a result, machine learning (ML), which is a subfield of artificial intelligence (AI), is catching on to assist FC in resolving its shortcomings. Using ML to enhance FC applications and deliver efficient services in terms of accuracy, latency reduction, energy consumption, security, privacy, resource, and traffic management [25,172,173] has been increasingly popular in recent times. Fog computing resource management involving ML enhances the computer, decision-making, and resource provisioning, along with delay prediction. Deploying ML techniques in fog computing facilitates accurate data processing and analyses in real-time while managing the network overhead as well as communication traffic, owing to fog’s decentralized model. The security aspects for the device, network, and data involving fog computing accompanied by ML prove to be effective. The merging of the fog model with machine learning has evolved into robust end-user and upper-layer services, allowing for deeper analytics and intelligent answers to tasks.

Machine learning (ML) is a promising option for intelligent data processing and inference and is a prime enabler to various IoT application domains [166], such as healthcare, smart home, smart agriculture, smart industry, smart grid, etc. It has a crucial part in designing the intelligent/smart setting for autonomous operations [167]. Machine learning has immense potential as a significant IoT technology gaining traction to provide insights for IoT applications [174]. IoT has excellent prospects for enhancing human life and industrial growth as innumerable sensing devices perform monitoring and increase communication potential [175]. For resource-constrained IoT devices, the confluence of machine learning with the cloud, edge, and fog is vital for IoT implementation [156,175] to usher in efficient performance, greater controllability, productivity, and cost reduction possibilities, while managing IoT’s QoS challenges.

Enabling intelligence at fog and IoT improves the overall performance [100]. FC moves the cloud’s potential to the edge of the network, where IoT and human users are present. Intelligence can be incorporated into FC as device-driven or human-driven. In a device-driven approach, fog and IoT are equipped with more sensing, processing, network, and storage capabilities, enabling context awareness for decision making and local resource management. In a human-driven model, human users act as the data source to the system, whose behavioral pattern is the key in shaping the network while serving them. Collectively, these two approaches can help meet IoT’s demand for QoS when designing fog computing systems.

The harnessing of machine learning in an IoT setting facilitates deeper analytics and helps materialize efficient and smart IoT applications [174]. Moreover, it can be utilized to overcome networking difficulties pertaining to routing, resource allocation, traffic engineering, and security [176,177,178,179,180]. Neural networks are deployed to effectively analyze enormous data produced by IoT [181]. Moreover, advanced AI involving deep learning has been thriving in data analytics, decision making, and prediction [85].

The potential of IoT has remarkably expanded thanks to the convergence of machine learning and artificial intelligence. Advanced machine intelligence approaches have enabled substantial insights into a number of real-world situations and the capacity to determine critical operational choices from the massive volume of IoT sensory data. As a result, ML and IoT must work in tandem to solve complicated real-world issues and fulfill computation and communication needs.

## 7. Conclusions

Cloud computing has revolutionized device interactions on the internet, which ushered in the Internet-of-Things and implemented a plethora of connected gadgets, with the potential to continually sense and respond to user requirements. The proliferation of networked IoT devices and ensuing big data and the rigorous demands of emerging IoT applications, such as low latency, location awareness, and mobility support in a geo-distributed scenario, have challenged the conventional cloud computing architecture. Hence, various computing paradigms such as edge and fog have emerged to address these limitations by deploying resources at the network’s edge. The computing at edge and fog implies collecting, processing, and analyzing data close to the data source and transmitting refined results to the centralized cloud, favoring time-sensitive applications that require increased accuracy, low latency, high-speed analytics, faster response time, improved reliability, and availability. Combining fog/edge with cloud computing has the prospect of aiding IoT in multiple ways. Because the fog and edge computing paradigms are up-and-coming, exhaustive research on this new technology is imperative. The evolving computing paradigms, as well as the challenges and opportunities, were explored in this study. Budding researchers can largely benefit from this extensive survey to comprehend recent advances in evolving computing paradigms.

## Figures and Tables

**Table 1:** List of acronyms used in the manuscript and their expansion.

Acronym	Full Form
AI	Artificial Intelligence
AR	Augmented Reality
CoT	Cloud of Things
CC	Cloud Computing
CCTV	Closed-Circuit Television
CPU	Central Processing Unit
CR	Cloud Robotics
DDoS	Distributed Denial of Service
DL	Deep Learning
EC	Edge Computing
ETSI	European Telecommunications Standard Institute
FaaS	Function-as-a-Service
FC	Fog Computing
IaaS	Infrastructure-as-a-Service
ICT	Information and Communications Technology
IDC	International Data Corporation
IoT	Internet-of-Things
IT	Information Technology
ITS	Intelligent Transport System
ITU	International Telecommunication Union
MACC	Mobile Ad hoc Cloud Computing
MEC	Multi-access Edge Computing
MC	Mobile Computing
MCC	Mobile Cloud Computing
MDC	Micro Data Center
mist	Mist Computing
ML	Machine Learning
MMA	Man-in-the-Middle Attack
MIT	Massachusetts Institute of Technology
NIST	National Institute of Standards and Technology
OP	Operational Technology
PaaS	Platform-as-a-Service
PRISMA	Preferred Reporting Items for Systematic Reviews and Meta-Analyses
QoE	Quality of Experience
QoS	Quality of Service
RAN	Radio Access Network
RAS	Reliability Availability Serviceability
SaaS	Software-as-a-Service
SDN	Software-Defined Networking
SLA	Service-Level Agreement
VM	Virtual Machine
VR	Virtual Reality
WSN	Wireless Sensor Network

**Table 2:** Review/survey papers and their contributions.

Author and Year	Articles Referenced	Time Span	Systematic Study	Survey/Review Outline	Computing Paradigms			Future Directions
					Cloud Computing	Fog Computing	Edge Computing	
Atzori et al. [14], 2016	119	1999 – 2016	×	The survey examines the prospect of Internet-of-Things from the evolutionary perspective, the role of IoT in modern society and ensuing challenges.	✓	×	✓	×
Hu et al. [15], 2017	123	2001 – 2017	×	The review presents fog computing features, architecture, compares with other computing paradigms, and summarizes key technologies that aid in application and deployment.	✓	✓	✓	✓
Mahmud et al. [16], 2017	47	2012 – 2016	×	The work presents a taxonomy from a comprehensive analysis of fog features and challenges pertaining to the structure, service, and security and identifies research gaps.	✓	✓	✓	✓
Lin et al. [17], 2017	167	2001 – 2017	×	The article offers a comprehensive overview of state-of-the-art IoT-enabling technologies, system architecture, privacy, security issues, and concerns of IoT and fog/edge computing integration during real-world deployment.	×	✓	✓	✓
Mao et al. [18], 2017	242	2003 – 2017	×	An exhaustive outline of state-of-the-art MEC from a communication viewpoint, resource management, comparison with MCC is presented.	✓	×	✓	✓
Naha et al. [19], 2018	142	2001 – 2018	×	The survey article presents fog computing overview, architecture, related technologies, taxonomy by analyzing fog requirement and reviewing challenges, research issues, and trends.	✓	✓	✓	✓
Mouradian et al. [20], 2018	168	2006 – 2017	✓	An exhaustive survey is tendered on fog computing architectures, algorithms, affiliated concepts, and their dissimilarities; additionally, challenges and research directions were discussed.	×	✓	×	✓
Mukherjee et al. [21], 2018	225	1997 – 2017	×	The survey extends an overview of fog computing basics, architecture and highlights the approach for service and allocation of resources to overcome latency, bandwidth, and energy consumption.	×	✓	×	✓
Elazhary [22], 2018	412	1991 – 2018	×	The exhaustive review researches arenas such as IoT, cloud computing, mobile computing, and related concepts and attempts to disambiguate emerging paradigms as well as technologies.	✓	✓	✓	✓
Atlam et al. [23], 2018	63	2012 – 2017	×	This work reviews fog computing state-of-the-art, including fog features, architecture, and merits, and insists on fog being an IoT enabler.	×	✓	×	✓
Bangui et al. [24], 2018	114	2012 – 2018	×	The review outlines edge computing technology and the challenges and concerns that accompany Distributed environments while shifting services from cloud’s centralized to edge’s decentralized platforms.	×	✓	✓	✓
Yousefpour et al. [2], 2019	450	2001 – 2018	×	A comprehensive survey is furnished that emphasizes fog computing, associated computing paradigms, and presents a taxonomy of research subjects, underlying challenges, and future leanings of fog.	✓	✓	✓	✓
Abdulkareem et al. [25], 2019	95	2011 – 2019	×	This review highlights recent advancements of ML techniques related to the accuracy, resource management and security of fog computing and its role in edge computing.	×	✓	✓	✓
Donno et al. [9], 2019	71	2004 – 2019	×	The review article offers clarification for beginners into research on cloud computing, edge computing, and fog computing by illustrating features and architecture of each paradigm and concludes by stating fog computing’s relevance as fog binds cloud, edge computing, and IoT together.	✓	✓	✓	✓
Khan et al. [26], 2019	101	2009 – 2019	×	The study focuses on cloud and state-of-the-art edge computing concepts, critical requirements, limitations and identified unaddressed issues.	✓	✓	✓	✓
Cao et al. [27], 2020	62	2005 – 2020	×	The article reviews research related to edge computing, summarizes key concepts, technologies, architecture, privacy, and security.	✓	×	✓	✓
Habibi et al. [28], 2020	191	2002 – 2019	×	The survey covers existing computing paradigms and emphasizes fog computing research areas by presenting a taxonomy and analyses from fog’s architectural viewpoint.	✓	✓	✓	✓
Moura et al. [29], 2020	194	1999 – 2020	×	This work surveys state-of-the-art fog computing systems, offers insights into designing and managing resilient fog systems and illustrates research issues and upcoming future trends.	×	✓	×	✓
Aslanpour et al. [30], 2020	50	2010 – 2020	×	The study offers a taxonomy of real-world performance metrics to assess the computing paradigms of cloud, fog, and edge.	✓	✓	✓	✓
Alli et al. [31], 2020	102	2009 – 2020	×	The article delves into the ecosystems of IoT–fog–cloud, analyzing concepts, architecture, standards, tools of fog Cloud-of-Things, and presents a taxonomy on emerging issues. It concludes that ML and AI in fog ecosystems would be appropriate for latency-sensitive and resource-constrained systems.	✓	✓	✓	✓

(✓: Yes, ×: No).

**Table 3:** Comparison of fog, edge and cloud computing characteristics.

Characteristic	Fog	Edge	Cloud
Operators	Users and cloud provider	Local enterprise or network infrastructure providers	Cloud provider
Participating Nodes	Fog devices (switches, routers, access points, etc.) and IoT devices	Edge devices	Fewer nodes spanning cloud to IoT devices
Service Type	Less global	Local	Global
Management	Distributed/centralized	Local business and service provider	Centralized
Hardware	Devices with virtualization facility (access points, routers, switches, servers)	Edge devices with compute capacity	Massive data centers and equipment with virtualization potential
Computation Device	Any device capable of computation, networking, and storage	Edge devices	Powerful cloud servers
Available Computing Resources	Moderate	Moderate	High
Nature of Failure	Highly diverse	Highly diverse	Predictable
Main Driver	Academia/ Industry	Academia/industry	Academia/industry
User Connectivity	Mostly wireless	Mostly wireless	High speed (Both wired and wireless)
Distance from Users	Relatively close	Close	Far
Internal Connectivity	Operate autonomously with intermittent or no internet connectivity	Operate autonomously with intermittent or no internet connectivity	Requires internet connectivity throughout service duration
Main Standardization Entity	OpenFog Consortium, IEEE	-	National Institute of Standards and Technology (NIST), Cloud Security Alliance (CSA), Distributed Management Task Force (DMFT), Open Commons Consortium (OCC), Global Inter-Cloud Technology Forum (GICTF)
Power Source	Battery/green energy/ direct power	Battery/green energy/direct power	Direct power
Power Consumption	Low	Low	High
Application Type	High computation with lower latency	Low latency computation	Ample computation
Architecture	Decentralized/hierarchical	Localized/distributed	Centralized/hierarchical
Computation Capacity	Moderate	Moderate	High
Storage Capacity	Limited	Limited	Massive storage capacity
Availability	High	Average	High
Latency	Low	Low	Relatively high
Node mobility	High	High	Very low
Security/Vulnerability	Must be provided on participant nodes	Must be provided on edge devices	Must be provided along Cloud-to-Things continuum
Server Location	Can be deployed at edge or dedicated locations	Near edge devices	Stationed in huge dedicated buildings
Number of Intermediate Hops	One/few	One	Multiple
Hardware Connectivity	WAN, LAN, WLAN, Wi-Fi, cellular	WAN, LAN, WLAN, Wi-Fi___33, cellular, ZigBee	WAN
Application Handling—real-time	Achievable	Achievable	Difficult owing to increased latency
Service Access	Through connected devices from the edge to the core	At the edge of the internet	Through core
Computation Cost	Low	Low	High
Cooling Cost	Very low	Very low	High
Deployment Space	Less	Less	Massive
Delay Cost	Less	Less	More

**Table 4:** Fog, edge, and cloud computing functionalities.

Feature	Fog	Edge	Cloud
Heterogeneity support	Yes	Yes	Yes
Connection to cloud	Yes	Yes or No	Yes
Infrastructure need	Yes	Yes	Yes
Geographically distributed	Yes	Yes	No
Virtualization technology	Yes	No	Yes
Location awareness	Yes	Yes	No
Ultra-low latency	Yes	Yes	No
Scalability	Yes	Yes	Yes
Mobility support	Yes	Yes	No
Application support—real-time	Yes	Yes	No
Application support—large-scale	Yes	Yes	Yes
Standardized	Yes	Yes	Yes
Multiple IoT applications	Yes	No	Yes
Data persistence	Yes	No	Yes
Computation migration	Yes	No	No
Conserving energy	Yes	Yes	No

**Table 5:** Open challenges and future research directions—summary.

Open Issue	Limitations Prevalent	Potential Solutions or Research Prospect	Related Specifics	Impact
Standardization of fog computing	Several fog definitions and related concepts are being proposed.	Formulate fog definition that can be universally accepted.	Foundation	Standards and Definition
Scalability	Major fog system schemes in practice fail to scale IoT vastitude.	Design algorithms and procedures that ensure scalability.	Scalability	Placement; Service Provisioning; Scheduling; Load Balancing; Offloading
Bandwidth-aware system	Although reducing bandwidth usage is key, fewer fog computing regard conserving bandwidth through fog systems.	Deliberate on saving bandwidth through fog systems and measure bandwidth usage under fog systems.	Bandwidth	Testbeds and Experiments; Control and Monitoring; Infrastructure Design
SLA for fog system	SLAs for cloud system are defined, but SLAs for fog systems are not defined.	Devise new SLA compatible for fog computing systems that supports multi-vendors.	Cost, QoS	Fog Infrastructure; Control and Monitoring
Mobility	Major existing work considers fixed fog nodes and mobile IoT devices.	Propose fog systems with mobile fog nodes and design suitable task offloading and scheduling plans ensuring availability to IoT nodes.	Mobility, Management	Concepts and Framework; Security and Privacy; Scheduling, Load Balancing and Offloading
Fog node site selection	The issue of site selection for fog node is highlighted by limited studies. The placing of fog servers at appropriate positions is crucial to offer maximum service. Analysis of demand and workload of a specific node prior to placement minimizes maintenance cost.	Devise site selection policies for fog nodes, addressing computation, communication, storage, and cost.	QoS, Cost, RAS	Resource Analysis and Estimation; Infrastructure Design
SDN support	Fog computing does not provide native support to SDN.	Improving and standardizing SDN for fog systems.	Programmability	Software and Tools; Definition and Standards
Resource Monitoring	Fog resource monitoring is addressed by very few studies.	Formulate procedures that monitor resources of fog systems involving multi-operators.	Management, Programmability	Software and Tools; Control and Monitoring
High-speed user support	Existing communication protocols do not assist high-speed users.	Develop protocols supporting high-speed users and mobility-predicting algorithms based on machine learning.	Mobility	Architecture and Framework
Federation	Federation schemes or application for fog is unavailable.	Formulate new fog node federation strategy operating across diverse domains.	Programmability, Management	Software and Tools
Fog node security	The fog nodes positioned at proximity of end user incites security challenge.	Configure secure fog nodes with robust access control policies that handle site attacks and secure hardware design to withstand physical damage.	Security, Device Heterogeneity	Security and Privacy; Hardware Design
Trust and authentication	Heterogeneous IoT nodes and fog nodes make the traditional authentication and trust strategies inept. The providers of fog service may be internet service provider, cloud vendor, or end-users, which jeopardizes the trust in fog.	Design of novel trust and authentication structure for user, service, and nodes is needful.	Heterogeneity, Security	Security and Privacy; Definition and Standards
Security for fog offloading	Fog node task offloading may lead to security and privacy concern.	Devise secure offloading technique and integrity, correctness checking scheme for task offloaded.	Security, QoS	Offloading, Security, and Privacy
Privacy	With various networks involved and fog operating predominantly on wireless technology, privacy issues arise. The end user can access numerous fog nodes which involves sensitive data.	Maintaining the privacy of sensitive personal data is vital.	Privacy	Privacy and Security
Flexibility	Fault or failure at network is not regarded by existing fog networks, with fog nodes being more prone to DoS attacks due to limited resources.	Regard fault prevention, detection, and recovery in fog networks and design DoS-resilient fog system.	Security	Security and Privacy; Infrastructure Design; Control and Monitoring
Green fog computing	Enhancing energy efficiency of overall fog system has to be deliberate.	Utilize battery storages and energy harvesting for IoT sensors and devices and place fog nodes near renewable energy sources.	Energy	Resource analysis, Estimation; Infrastructure Design
Energy consumption	With huge number of fog nodes, energy consumed is large. The energy demand of fog nodes should be reduced to mitigate cost and energy.	Device resource provisioning strategy that is energy efficient, while being aware of fog node positions.	Energy	Resource Analysis, Estimation; Infrastructure Design
Multi-objective design	Many existent schemes reckon certain objectives and overlook other objectives.	Propound schemes that regard multiple objectives concurrently (task offload strategy that deems availability, bandwidth, energy, and security).	QoS	Scheduling, Load Balancing, and Offloading; Resource Analysis and Estimation; Testbeds and Experiments

## Data Availability

Not applicable.
